# Comprehensive Metabolomics Identified the Prominent Role of Glycerophospholipid Metabolism in Coronary Artery Disease Progression

**DOI:** 10.3389/fmolb.2021.632950

**Published:** 2021-04-14

**Authors:** Hui Chen, Zixian Wang, Min Qin, Bin Zhang, Lu Lin, Qilin Ma, Chen Liu, Xiaoping Chen, Hanping Li, Weihua Lai, Shilong Zhong

**Affiliations:** ^1^ Guangdong Provincial People’s Hospital, Guangdong Academy of Medical Sciences, School of Medicine, South China University of Technology, Guangzhou, China; ^2^ Department of Pharmacy, Guangdong Provincial People’s Hospital, Guangdong Academy of Medical Sciences, Guangzhou, China; ^3^ Guangdong Provincial Key Laboratory of Coronary Heart Disease Prevention, Guangdong Cardiovascular Institute, Guangdong Provincial People’s Hospital, Guangdong Academy of Medical Sciences, Guangzhou, China; ^4^ School of Biology and Biological Engineering, South China University of Technology, Guangzhou, China; ^5^ Department of Cardiology, Guangdong Provincial People’s Hospital, Guangdong Academy of Medical Sciences, Guangzhou, China; ^6^ Department of Cardiology, Xiangya Hospital, Central South University, Changsha, China; ^7^ Department of Cardiology, the First Affiliated Hospital, Sun Yat-sen University, Guangzhou, China; ^8^ Department of Clinical Pharmacology, Xiangya Hospital, Central South University, Changsha, China

**Keywords:** coronary artery disease, metabolome, lipidome, severity, glycerophospholipid metabolism, diagnostic marker

## Abstract

**Background:** Coronary stenosis severity determines ischemic symptoms and adverse outcomes. The metabolomic analysis of human fluids can provide an insight into the pathogenesis of complex disease. Thus, this study aims to investigate the metabolomic and lipidomic biomarkers of coronary artery disease (CAD) severity and to develop diagnostic models for distinguishing individuals at an increased risk of atherosclerotic burden and plaque instability.

**Methods:** Widely targeted metabolomic and lipidomic analyses of plasma in 1,435 CAD patients from three independent centers were performed. These patients were classified as stable coronary artery disease (SCAD), unstable angina (UA), and myocardial infarction (MI). Associations between CAD stages and metabolic conditions were assessed by multivariable-adjusted logistic regression. Furthermore, the least absolute shrinkage and selection operator logistic-based classifiers were used to identify biomarkers and to develop prediagnostic models for discriminating the diverse CAD stages.

**Results:** On the basis of weighted correlation network analysis, 10 co-clustering metabolite modules significantly (*p* < 0.05) changed at different CAD stages and showed apparent correlation with CAD severity indicators. Moreover, cross-comparisons within CAD patients characterized that a total of 72 and 88 metabolites/lipid species significantly associated with UA (vs. SCAD) and MI (vs. UA), respectively. The disturbed pathways included glycerophospholipid metabolism, and cysteine and methionine metabolism. Furthermore, models incorporating metabolic and lipidomic profiles with traditional risk factors were constructed. The combined model that incorporated 11 metabolites/lipid species and four traditional risk factors represented better discrimination of UA and MI (C-statistic = 0.823, 95% CI, 0.783–0.863) compared with the model involving risk factors alone (C-statistic = 0.758, 95% CI, 0.712–0.810). The combined model was successfully used in discriminating UA and MI patients (*p* < 0.001) in a three-center validation cohort.

**Conclusion:** Differences in metabolic profiles of diverse CAD subtypes provided a new approach for the risk stratification of unstable plaque and the pathogenesis decipherment of CAD progression.

## Introduction

Coronary artery disease (CAD) refers to underlying coronary artery atherosclerotic lesions that cause vascular lumen stenosis or occlusion and insufficient blood supply and result in myocardial ischemia, hypoxia, or necrosis (Malakar et al., 2019). Despite the advances in medical treatment, percutaneous coronary intervention, and surgical therapies, atherosclerotic CAD persists as a major clinical problem leading to a significant proportion of mortality of aging populations (Zhou et al., 2019; Virani et al., 2020). CAD can be stratified into stable coronary artery disease (SCAD), unstable angina (UA), and myocardial infarction (MI) according to the clinical symptoms, the extent of arterial blockage, and the condition of myocardial damage (Shao et al., 2020). Atherosclerotic plaque accumulation and development become chronic, complicated, and dynamic over time. The detailed mechanisms of plaque formation and development are poorly known. Thus, novel biomarkers for patients with risks of plaque instability and rupture need to be identified to delay onset and improve treatment.

Emerging metabolomics is a powerful tool to systematically investigate the functional small molecule in biological fluid samples. An abnormal metabolome can reportedly characterize CAD, further providing clues for physiological and pathological explorations (Wishart, 2016; Tzoulaki et al., 2019). Elevated plasma trimethylamine N-oxide levels can predict a future risk of major adverse cardiac events (MACE) and an increased prevalence of cardiovascular disease (CVD) (Dannenberg et al., 2020; Gencer et al., 2020). Short-chain fatty acids and primary and secondary bile acids affect CVD progression (Fan et al., 2016; Tang et al., 2019). Previous studies highlighted the key role of lipid species in the formation and subsequent disruption of atherosclerotic plaques, including ceramides, sphingomyelin, phosphatidylcholines, and cholesterol esters (Wang D. D. et al., 2017; Poss et al., 2020). Altered lipid metabolism correlated with inflammation and oxidative stress, such as the oxidation of phospholipids and cholesterol in LDL and played an important part in the formation of lipid-laden foam cells within the intima to the necrotic lipid core of unstable plaque (Meikle et al., 2011; Lu et al., 2017; Zhong et al., 2019). However, the relationship between plasma metabolic profiling and detailed characterization and quantification of atherosclerosis burden at different CAD stages needs to be systematically elucidated.

The goals of the present study were to comprehensively investigate the plasma metabolomic and lipidomic signatures associated with increased CAD severity and to evaluate the significantly differential metabolites and lipid species for their use in discriminating the subgroups of CAD, thereby providing an enhanced understanding of disease progression. Herein, we performed a widely targeted metabolomic and lipidomic evaluation in plasma of patients with SCAD, UA, and MI and identified specific features of metabolite profiles that are associated with increase in CAD severity and can be used to differentiate these three subgroups. The subsequent pathway analysis revealed that glycerophospholipid metabolism was the most significantly altered metabolic pathway. Disease diagnostic classifiers for discriminating between different CAD subgroups were constructed and validated based on novel metabolic markers and traditional risk factors.

## Materials and Methods

### Study Population

**FIGURE 1:**
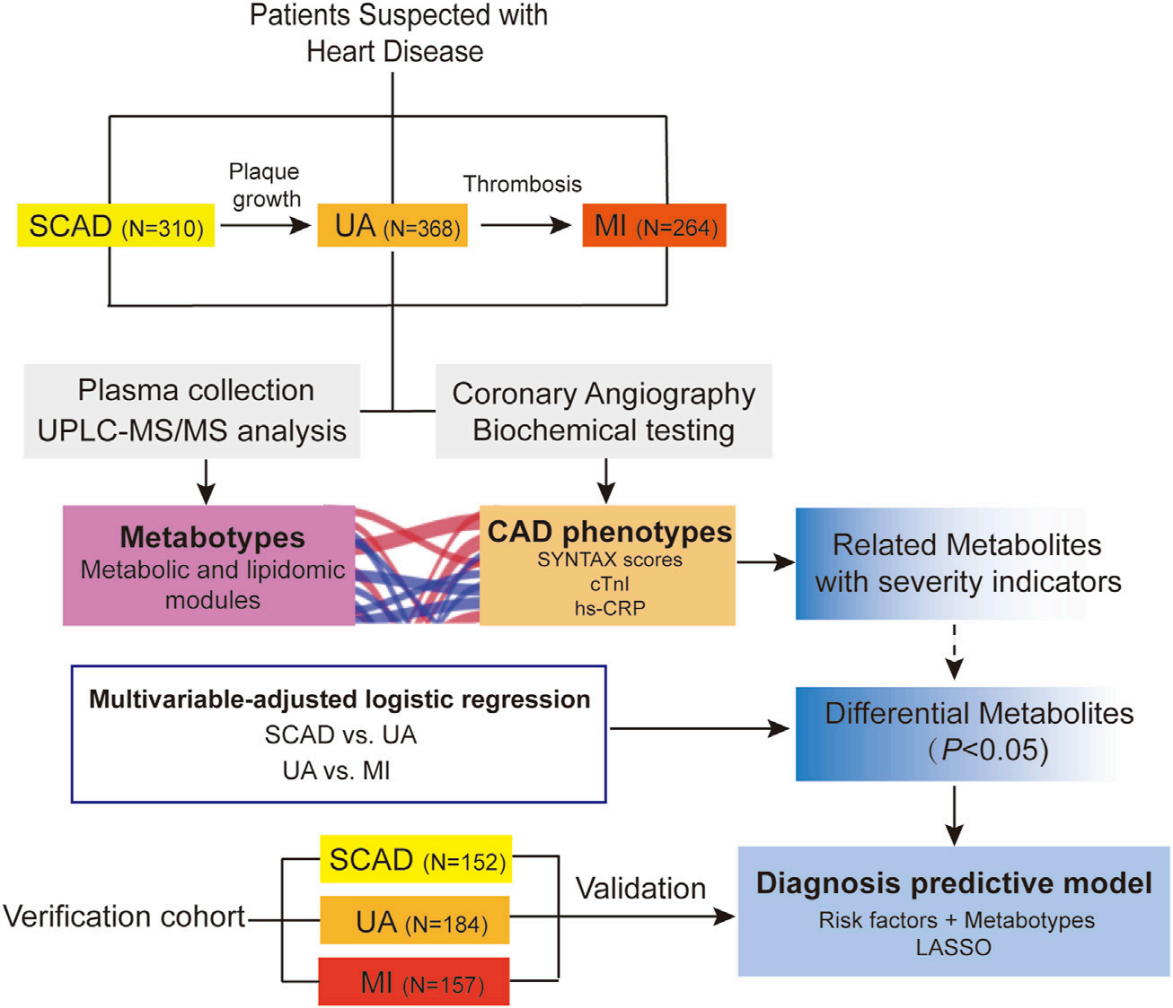
Overview of workflow chart for data generation and analysis. CAD, coronary artery disease; cTnI, cardiac troponin I; hs-CRP, high-sensitivity C-reactive protein; LASSO, least absolute shrinkage and selection operator; MI, myocardial infarction; SCAD, stable coronary disease; UA, unstable angina; SYNTAX scores, Synergy between percutaneous coronary intervention with TAXUS and Cardiac Surgery scores; UPLC-MS/MS, ultra-performance liquid chromatography-tandem mass spectrometry.

An overview of the workflow is depicted in Figure 1. This study was a two-stage study that included a total of 1,435 Chinese subjects with CAD. In the discovery cohort (*N* = 942), we evaluated the association of plasma metabolome and lipidome with CAD using consecutively enrolled samples with clinical and demographic information obtained from Guangdong Provincial People’s Hospital (Cai et al., 2018) in 2010–2014. In the verification cohort (*N* = 493), we enrolled multi-center patients with CAD from three centers (including Guangdong Provincial People’s Hospital, Xiangya Hospital of Central South University, and the First Affiliated Hospital of Sun Yat-sen University) from 2017 to 2018.

All subjects were 18–80 years and met the diagnostic criteria of CAD. They were further stratified into three subgroups (SCAD, UA, and MI) on the basis of a detailed diagnosis performed by cardiologists, their symptoms, ischemic changes in electrocardiogram, laboratory measurements, and coronary angiographic results. The specific diagnostic criteria of CAD subtypes are summarized under Supplemental Materials: Supplementary Methods. The exclusion criteria were as follows: 1) severe renal dysfunction, serum creatinine >3.0 mg/dl, renal transplantation, or dialysis; 2) liver dysfunction, alanine aminotransferase >135 U/L, or cirrhosis; 3) during pregnancy or lactation; 4) malignant tumors or hemodialysis; 5) autoimmune disorders; and 6) unavailable information. Demographic information, medication history, and biochemical measurements were collected according to standard procedures and obtained from the hospital electronic case system.

### Ethics Statement

The study fully complies with the guidance of the Helsinki Declaration. The Medical Ethical Review Committee of Guangdong Provincial People’s Hospital granted ethics approval (GDREC2010137 and GDREC2017071H). Written informed consent was obtained from all subjects.

### Plasma Sample Collection

Each eligible subject fasted for at least 8 h to minimize the influence of nutrition on metabolite levels. The subjects’ venous blood samples were collected in ethylene diamine tetraacetic acid (EDTA) vacutainer tubes in the morning (between 9 AM and 12 PM) after overnight fasting and cooled in a freezer (4°C) immediately. Plasma was separated by centrifugation (2095 *g*, 10 min, 4°C) within 2 h and refrigerated at −80°C until analysis.

### Severity Evaluation of Coronary Artery Disease Via Angiographic Analysis

Coronary angiography (CAG) was performed to define the extent and severity of CAD in patients with suspected symptoms whose clinical characteristics and results of noninvasive testing indicated a high likelihood of CAD and who are amenable to, and candidates for coronary revascularization (Fihn et al., 2014). CAG was performed using the standard technique and images of coronary angiograms were obtained from Syngo Dynamics cardiovascular imaging software (Siemens Medical Solutions, United States, Inc, Malvern, Pennsylvania). The complexity and burden of atherosclerotic CAD were evaluated using an angiographic scoring system (SYNTAX scores) (Thuijs et al., 2019; Takahashi et al., 2020) and diagnosed by two professional cardiologists blinded to the clinical outcome (details are presented in the Supplemental Materials: Supplementary Methods).

### Widely Targeted Metabolomic Analysis and Data Preprocessing

The hydrophilic and hydrophobic compounds were extracted from each plasma sample and detected via ultra-performance liquid chromatography and electrospray ionization-tandem mass spectrometry (UPLC-ESI-MS/MS) system in the positive and negative ionization modes in Metware Biotechnology (Wuhan, China). Details for the sample preparation and UPLC-MS/MS experiment parameters are provided in the Supplemental Materials: Supplementary Methods.

In total, 202 metabolites (including nucleosides, hormones, carbohydrates, organic acids and derivatives, and amino acids and derivatives) and 667 lipid species (including ceramides, cholesteryl esters, diacylglycerol, lysophosphatidic acid, lysophosphatidylcholine, lysophosphatidylethanolamine, lysophosphatidylserine, monoglyceride, phosphatidic acid, phosphatidylcholines, phosphatidylglycerol, phosphatidylserine, phosphatidylethanolamine, and triacylglycerol) were identified and quantified.

Quality control (QC) samples were utilized for the normalization of the data. A QC sample was created via pooling aliquots from all samples and was injected every 10 samples throughout the run to assess the instrument’s stability. Highly stable QC data showed that the run had great repeatability and reliability (Supplementary Figure S1).

For metabolomic and lipidomic analyses, raw signals with more than half of the missing rate in the QC samples (those with zero ion intensity) were removed. Missing metabolomic data were imputed by replacing the missing value with a minimum value of the metabolite quantified. To adjust signal drift, we applied the Quality Control–based Robust LOESS (LOcally Estimated Scatterplot Smoothing) Signal Correction (QC–RLSC) algorithm for analytical batch effect correction (Luan et al., 2018), which is an effective way to normalize the metabolic features to the QC samples within an analytical block. The dataset of discovery cohort after batch effect correction is available in Supplemental Materials: Supplementary Table S3. The dataset was then scaled by pareto scaling with procedures of mean centering and scaling to the square root of standard deviation (van den Berg et al., 2006). Then, the matrix was exported for further analysis.

### Clustering of Metabolites Using Weighted Correlation Network Analysis.

A metabolic network was constructed by the weighted correlation network analysis (WCNA), which used metabolites’ pairwise correlations to identify modules of highly correlated metabolites (Pei et al., 2017). An unsigned weighted metabolite co-expression network was constructed. Considering the scale-free topology fit index and mean connectivity, the soft-thresholding power *β* = 4 and min module size = 5 were chosen for the analysis. Spearman correlation between metabolite modules and clinical parameters was calculated using R. The Benjamini–Hochberg method was used to control the false discovery rate (FDR). Hub metabolites indicated a high degree of connectivity in biological interaction networks and ,thus, they were considered biologically important. Clusters of co-abundant plasma metabolites were identified using the “WGCNA” package in R.

### Statistical Analysis

Among the baseline characteristics of the study population, continuous variables were described using medians (interquartile ranges) and were compared using Mann–Whitney U tests (non-normal distribution). Categorical variables were presented as counts (percentages) and were compared with Chi-squared tests. Statistical significance was determined as *p* < 0.05.

The linear regression analysis, adjusted for traditional Framingham risk factors, including age, sex, hypertension, diabetes mellitus, smoking, low-density lipoprotein cholesterol (LDLC), high-density lipoprotein cholesterol (HDLC), and triglycerides (TG) (Senthong et al., 2016), was applied to examine the associations of metabolomic and lipidomic profiles with SYNTAX score, SYNTAX score Ⅱ, hs-CRP, and cardiac troponin I (cTnI) levels.

To assess the association of individual metabolomic and lipidomic signatures against the different stages of CAD, we performed adjusted logistic regression of metabolomic and lipidomic profiles against SCAD vs. UA and UA vs. MI to estimate the odds ratios (ORs) and 95% confidence intervals (CIs). To avoid potential confounders, traditional risk factors, including age, sex, hypertension, diabetes mellitus, smoking, LDLC, HDLC, and TG, were used as covariates for adjustment. Subjects with missing covariates were omitted. Statistical significance was determined as a *p*-value of <0.05. Open database sources, including the Kyoto Encyclopedia of Genes and Genomes (KEGG) databases (http://www.genome.jp/kegg/) and the MetaboAnalyst (https://www.metaboanalyst.ca) (version 4.0), were used to identify the highly enriched metabolic pathways based on the significantly differential levels of metabolites and lipid species.

In the development of diagnostic models to classify CAD subgroups, firstly, the following were added to develop the traditional risk factor-based model in a stepwise regression (forward and backward) with the aim to minimize the Akaike information criterion (AIC): age, sex, hypertension, diabetes mellitus, smoking, LDLC, HDLC, and TG (traditional risk factors); APOA and Lp(a) (risk lipid traits); and left ventricular ejection fraction (LVEF, heart function indicator). This procedure was performed within 10 iterations of a 5-fold cross-validation framework (“MASS”, “caret” packages). Subsequently, metabolites and lipid species that were nominally significantly (*p* < 0.05) associated with UA (vs. SCAD) and MI (vs. UA) in the adjusted logistic regression analysis were included into least absolute shrinkage and selection operator (LASSO) penalized models (“glmnet” package) to further reduce the number of markers and select the most powerful predictive features. In the LASSO selection analysis, the optimal value for the tuning parameter λ was determined via 5-fold cross-validation (200 iterations). We adopted the largest value of lambda, such that the error was within one standard error of the minimum, known as “1-se” lambda. The relative contribution of features to classification assignment (UA vs. SCAD and MI vs. UA) was determined by the occurrence frequency in our multivariate model training. The feature was selected with an occurrence frequency of more than 100 times.

To evaluate the predictability of the models, a binary logistic regression model was then fitted using the chosen biomarkers as the covariates; this model was generated as follows: combined diagnostic score (probability) = 1/1 + exp [-(intercept + coefficient1 (biomarker1) + coefficient2 (biomarker2) … + coefficient n (biomarker n))]. The area under the curve (AUC, equivalently known as C-statistic) of the receiver operating characteristic (ROC) was applied to calculate the proportions of concordant pairs among all pairs of observation with 1.0 indicating perfect prediction accuracy. Moreover, the continuous net reclassification improvement (NRI) and integrated discrimination improvement (IDI) were calculated in assessing the models. The 95% confidence intervals (CIs) were estimated for each parameter. The difference of combined diagnostic scores between CAD subgroups in the validation cohort was examined by the Wilcoxon rank sum test.

All the above analyses were conducted on the R platform (version 3.6.1, http://www.R-project.org/).

## Results

### Characteristics of the Study Population

A total of 1,435 CAD patients were included from three independent centers in China (Figure 1). The discovery cohort included 942 participants enrolled at Guangdong Provincial People’s Hospital, which were further classified into the following groups on the basis of the guidelines for diagnosis: SCAD (*N* = 310), UA (*N* = 368), and MI (*N* = 264). The baseline characteristics and laboratory data of each group are shown in Table 1. With disease shifting, the disturbance in lipid metabolism occurred with decreasing HDL-C and APOA but increasing Lp(a). Inflammatory state increased, as significant differences in hs-CRP levels were found between UA vs. MI (*p* < 0.001). The systemic atherosclerotic burden of CAD was determined using SYNTAX score system, and the median scores of each group were as follows: SCAD, 13.0 (8.0, 23.0); UA, 14.0 (9.0, 22.0); and MI, 19.0 (10.0, 27.1). The SYNTAX score showed a significant difference between SCAD vs. MI (*p* < 0.001) and UA vs. MI (*p* < 0.001). The MI group exhibited a higher proportion of three-stenosed vessels (37.56%), a larger left ventricular mass index (LVMI), and a lower LVEF. The median levels of cTnI, an indicator of myocardial infarction, were 0.01 (0.005, 0.04), 0.02 (0.008, 0.02), and 0.3 (0.04, 1.9) μg/ml in the SCAD, UA, and MI groups, respectively. Significant differences in cTnI levels were found with SCAD vs. UA (*p* = 0.025) and UA vs. MI (*p* < 0.001).

**TABLE 1:** Baseline characteristics of discovery cohort.

	SCAD (*N* = 310)	UA (*N* = 368)	MI (*N* = 264)	*p* Value	
				SCAD vs. UA	UA vs. MI
Age, years	63.2 (56.9, 70.4)	65.0 (57.5, 72.5)	61.6 (52.9, 68.9)	0.066	<0.001
Male	243 (78.4)	286 (77.7)	228 (86.4)	0.91	0.0081
SBP, mmHg	132 (120, 145)	130 (120, 143)	124 (110, 135)	0.37	<0.001
BMI, kg/m^2^	24 (22, 27)	24 (22, 26)	24 (21, 26)	0.2	0.16
Current smokers	79 (25.6)	98 (27.1)	97 (37.0)	0.74	0.01
**Comorbidities**					
Hypertension	200 (64.7)	235 (63.9)	129 (48.9)	0.88	<0.001
Hyperlipidemia	39 (12.6)	41 (11.1)	24 (9.1)	0.64	0.48
Arrhythmia	30 (9.7)	38 (10.3)	11 (4.2)	0.89	<0.001
Diabetes mellitus	83 (26.7)	100 (27.2)	75 (28.4)	0.99	0.80
**Laboratory data**					
ALT, U/L	22.0 (17.9, 29.0)	23.5 (18.0, 33.0)	28.0 (19.0, 39.0)	0.049	<0.001
AST, U/L	23.0 (19.0, 27.0)	24.0 (20.0, 29.0)	26.0 (21.0, 36.0)	0.064	<0.001
GLUC, mmol/L	5.6 (5.0, 7.1)	5.8 (5.0, 7.3)	6.0 (5.1, 7.9)	0.21	0.13
eGFR, ml/min/1.73 m^2^	90.1 (76.0, 106.5)	87.5 (70.8, 103.6)	87.7 (71.2, 103.0)	0.08	0.57
CK, U/L	89.5 (64.0, 122.0)	86.0 (63.0, 116.0)	86.0 (62.1, 132.8)	0.41	0.60
CKMB, U/L	5.9 (4.3, 8.2)	6.6 (4.7, 9.2)	6.9 (5.0, 9.2)	0.04	0.34
TC, mmol/L	4.1 (3.5, 5.0)	4.2 (3.5, 4.8)	4.0 (3.5, 4.7)	0.47	0.18
TG, mmol/L	1.3 (1.0, 1.9)	1.4 (1.0, 1.9)	1.3 (1.0 1.8)	0.29	0.51
LDLC, mmol/L	2.4 (1.9, 3.1)	2.5 (2.0, 3.1)	2.4 (1.9, 3.0)	0.73	0.52
HDLC, mmol/L	0.97 (0.84, 1.12)	0.94 (0.81, 1.12)	0.85 (0.72, 0.99)	0.1	<0.001
APOA, g/L	1.07 (0.90, 1.23)	1.01 (0.89, 1.21)	0.93 (0.80, 1.09)	0.11	<0.001
Lp(a), mg/dL	151.0 (76.0, 400.9)	169.4 (80.9, 357.3)	238.1 (118.9, 457.8)	0.98	0.0053
CREA, μmol/L	80.7 (69.0, 93.0)	81.4 (71.0, 97.0)	85.0 (73.5, 100.0)	0.21	0.026
BNP, pg/mL	114.4 (41.2, 278.0)	168.7 (59.9, 549.0)	670.1 (280.3, 1749.0)	0.0025	<0.001
hs-CRP, mg/L	2.3 (0.7, 4.5)	2.1 (1.0, 6.4)	6.4 (2.2,15.2)	0.14	<0.001
cTnI, μg/mL	0.01 (0.005, 0.04)	0.02 (0.008, 0.02)	0.3 (0.04, 1.9)	0.025	<0.001
**Medication**					
*ß*-blockers	277 (89.6)	319 (86.9)	239 (90.5)	0.33	0.20
ACEI or ARB	191 (61.8)	225 (61.3)	182 (68.9)	0.91	0.058
CCBs	95 (30.7)	113 (30.8)	49 (18.6)	1	<0.001
PPIs	152 (49.2)	176 (48.0)	127 (48.1)	0.81	1
Cardiac function					
SYNTAX score	13.0 (8.0, 23.0)	14.0 (9.0, 22.0)	19.0 (10.0, 27.1)	0.76	<0.001
SYNTAX score Ⅱ	26.0 (22.0, 32.0)	27.0 (21.0, 34.0)	28.0 (22.0, 34.0)	0.09	0.57
Counts of Long-lesion				0.53	0.0017
1	74 (23.9)	87 (23.6)	97 (36.7)		
2	25 (8.1)	30 (8.2)	23 (10.2)		
3	5 (1.6)	2 (0.5)	3 (1.1)		
4	-	1 (0.3)	-		
No. of SV				0.032	0.067
1	75 (26.8)	118 (35.0)	61 (25.3)		
2	110 (39.3)	109 (32.3)	81 (33.6)		
3	77 (27.5)	99 (29.4)	90 (37.3)		
LVEF	65.0 (61.0, 69.0)	65.0 (60.0, 69.0)	54.0 (45.0, 63.0)	0.31	<0.001
LVMI	112.5 (97.9, 132.8)	112.7 (95.6, 135.8)	125.6 (103.7, 150.2)	0.73	0.0015

Data are shown as median (interquartile range) or n (%). p values were calculated using Mann–Whitney *U* test for non-normally distributed continuous variables and the Chi-squared test for categorical variables. ACEI, angiotensin converting enzyme inhibitor; ALT, alanine aminotransferase; APOA, apolipoprotein A; ARB, angiotensin receptor Blocker; AST, aspartate aminotransferase; BMI, body mass index; BNP, B-type natriuretic peptide; CCB, calcium channel blocker; CK, creatine kinase; CKMB, MB isoenzyme of creatine kinase; CREA, Creatinine; cTnI, cardiac troponin I; eGFR, estimated glomerular filtration rate; GLUC, glucose; HDLC, high-density lipoprotein cholesterol; hs-CRP, high-sensitivity C-reactive protein; LDLC, low-density lipoprotein cholesterol; Lp(a), lipoprotein(a); LVEF, left ventricular ejection fraction; LVMI, left ventricular mass index; MI, myocardial infarction; No of SV, No. of stenosed vessels; PPI, proton pump inhibitor; SBP, systolic blood pressure; SCAD, stable coronary artery disease; SYNTAX, Synergy between PCI with TAXUS and Cardiac Surgery; TC, total cholesterol; TG, triacylglycerol; UA, unstable angina.

The validation cohort from three centers included 493 participants. Their baseline characteristics are summarized in Supplementary Table S11.

### Identification of Modules Associated With Multiple Clinical Traits

In the WCNA, 756 of the metabolites and lipid species in the discovery set were parsed into 35 co-abundance modules, whereas the gray module comprised unassigned metabolites and lipids due to weak correlation with others. However, each metabolite and lipid were further analyzed individually.

**FIGURE 2:**
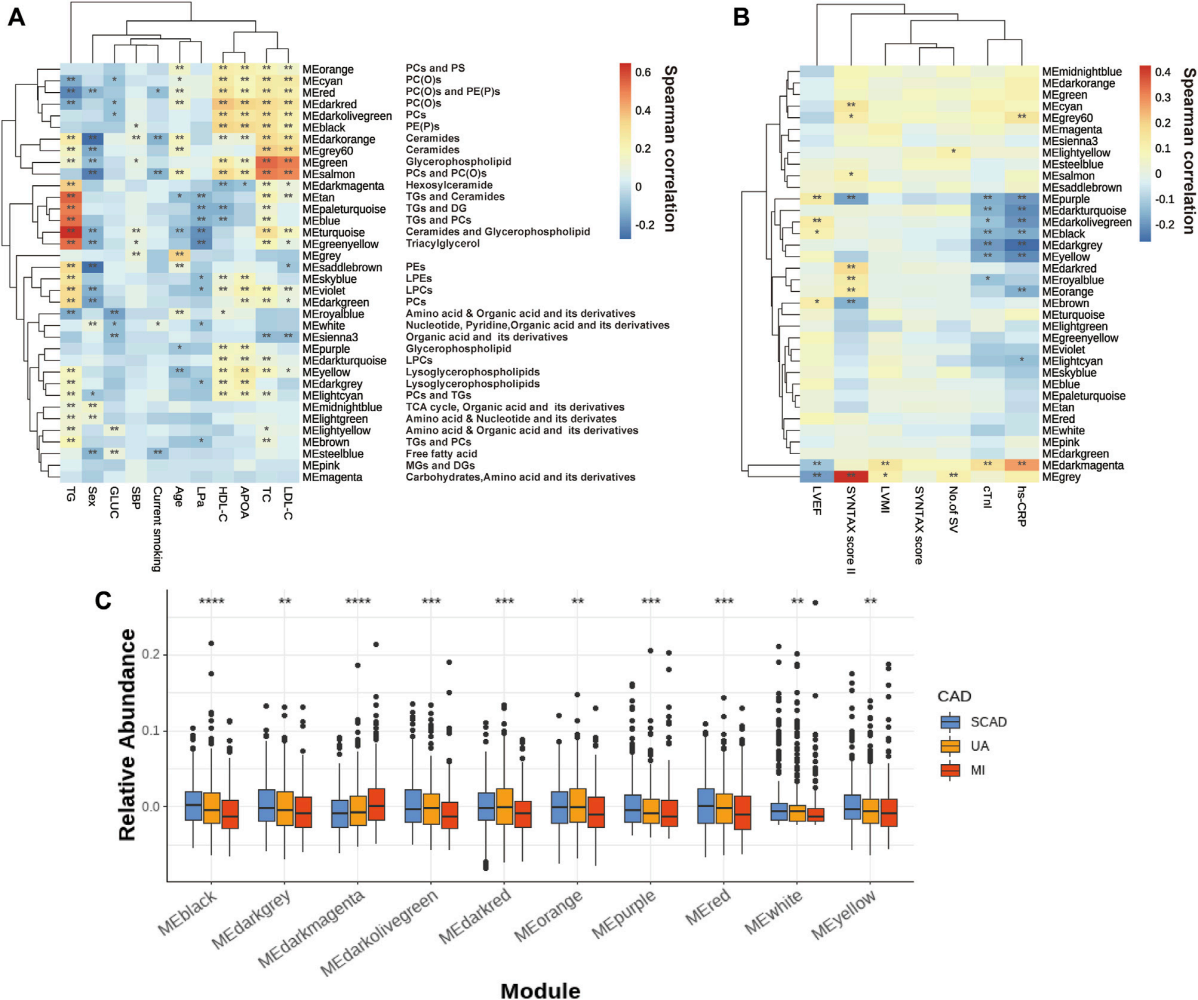
Assessment of the metabolite modules associated with the CAD progression. **(A)** Heatmap of metabolite modules and major CAD risk factors. **(B)** Heatmap of metabolite modules and major CAD phenotypes. **(C)** Boxplot of 10 significantly altered metabolite modules between CAD subgroups compared by the Wilcoxon rank sum test. In **(A,B)**, the colors varying from blue to orange indicate negative to positive correlations, and *FDR< 0.05, **FDR <0.01 by the spearman correlation. In **(C)**, boxes represent the inter-quartile ranges, lines inside the boxes denote medians and the asterisk represents *p* values < 0.05 by the Wilcoxon rank sum test. APOA, apolipoprotein A; GLUC, glucose; HDLC, high-density lipoprotein cholesterol; LDLC, low-density lipoprotein cholesterol; LP(a), lipoprotein(a); LVEF, left ventricular ejection fraction; LVMI, left ventricular mass index; No. of SV, No. of stenosed vessels; SBP, systolic blood pressure; TC, total cholesterol; TG, triacylglycerol; other abbreviations as in Figure 1.

The correlations of thirty-five eigenmetabolites of the modules and external traits are shown in Figures 2A,B (Detailed annotation and information are listed in Supplementary Tables S2–S5). We identified 16 of 35 modules (45.7%) that were significantly associated with major CAD phenotypes (either SYNTAX scores, number of stenosed vessels, LVEF, LVMI, or cTnI levels). Moreover, by abundance cross-comparison, 10 of these 35 modules (28.6%) showed significant differences with *p* < 0.05 between CAD stages (Figure 2C). Notably, the eigenmetabolites in darkmagenta module (hexocylceramides) were positively correlated with hs-CRP levels (Rho = 0.28, FDR <0.001), cTnI (Rho = 0.17, FDR = 0.0034), LVMI (Rho = 0.14, FDR = 0.0030), and TG (Rho = 0.35, FDR <0.001) but negatively correlated with LVEF (Rho = −0.12, FDR = 0.0077) and HDL-C (Rho = −0.12, FDR = 0.0095) (Figure 2A). Moreover, the dark gray module (lysoglycerophospholipids) was negatively correlated with hs-CRP (Rho = −0.27, FDR <0.001) and cTnI (Rho = −0.19, FDR <0.001) but positively correlated with APOA (Rho = 0.23, FDR <0.001), HDLC (Rho = 0.18, FDR <0.001), and TG (Rho = 0.17, FDR <0.001, Figure 2A).

Several modules also showed strong correlations with the conventional lipid traits (Supplementary Table S2). Except for the modules with triglyceride inside, sphingolipids such as Cer and glycerophospholipids such as PEs and LPCs showed a close correlation with TC, LDLC, HDLC, and APOA. For example, the black module contains PE(P)s and dark red module contains PC(O), both of which showed a decreasing tendency with disease development (Figure 2C); these were positively correlated with HDLC (Rho = 0.334, FDR = 1.20E-23; Rho = 0.371, FDR= 2.06E-29) and APOA (Rho = 0.318, FDR= 4.83E-18; Rho = 0.339, FDR= 1.28E-20). Moreover, green (glycerophospholipids) and gray60 (ceramides), were positively correlated with TC (Rho = 0.541, FDR = 3.84E-68; Rho = 0.360, FDR = 1.00E-27) and LDLC (Rho = 0.506, FDR = 1.93E-58; Rho = 0.336, FDR = 6.43E-24).

### Correlations Between Plasma Metabolite Levels and Severity Indicators

The linear regression analysis of metabolites and lipid species to the SYNTAX scores (atherosclerotic burden), cTnI (myocardial necrosis), and hs-CRP (inflammatory state) was conducted by adjusting for traditional risk factors, including age, sex, hypertension, diabetes mellitus, smoking, LDLC, HDLC, and TG. Numerous metabolites and lipid species showed strong association for one or more severity indicators (Supplementary Tables S6–S9). Three lipid species, namely, the hexosylceramide HexCer(d18:1/22:0) and the alkylphosphatidylcholine PC(O-32:0) and PC(O-42:3), were consistently significantly (*p* < 0.05) correlated with four severity indicators (Supplementary Figure S2A, Supplementary Tables S6–S9). We also found that high HexCer (d18:1/22:0) exhibited a high proportion of three-stenosed vessels (stenosed defined as >50%) with a univariate estimate of 0.11 ± 0.039, *p* = 0.0057, and an adjusted estimate (for the traditional risk factors above) of 0.082 ± 0.040, *p* = 0.042 (Supplementary Figure S2B).

### Changes in the Plasma Metabolomic Features Between Different Coronary Artery Disease Subgroups

**FIGURE 3:**
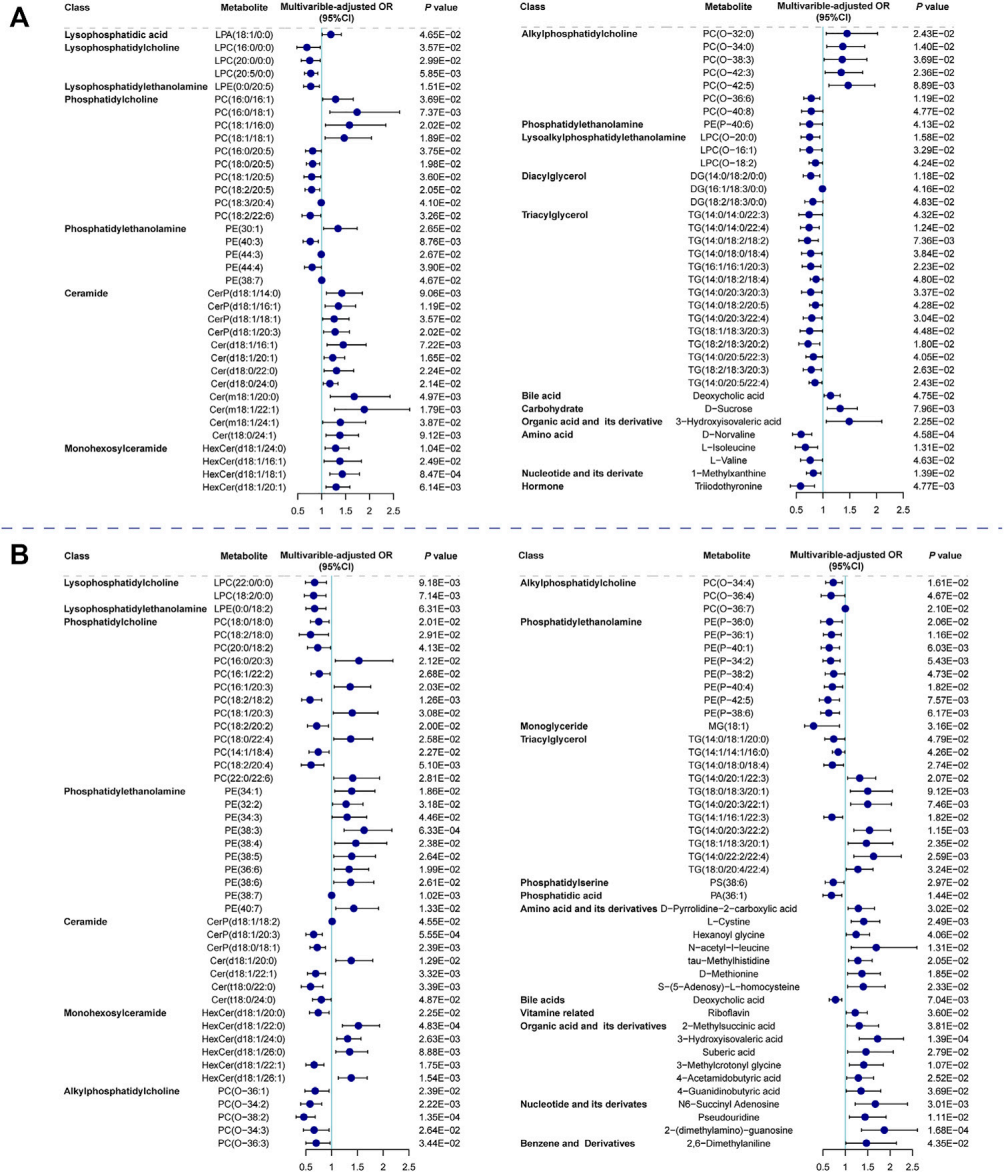
Relationship between metabolic features against UA (vs. SCAD) and MI (vs. UA). Forest plot of odds ratios and 95% confidence intervals for logistic regression of individual metabolites/lipid species against **(A)** SCAD vs. UA (*p* < 0.05) **(B)** UA vs. MI (*p* < 0.05), adjusting for age, sex, hypertension, diabetes mellitus, smoking, LDLC, HDLC and TG. Cer, ceramide; CI, confidence interval; DG, diacylglycerol; HexCer, hexosylceramide; LPA, lysophosphatidic acid; LPC, lysophosphatidylcholine; LPC(O), lysoalkylphosphatidylethanolamine; LPE, lysophosphatidylethanolamine; MG, monoglyceride; OR, odds ratio; PA, phosphatidic acid; PC, phosphatidylcholine; PC(O), alkylphosphatidylcholine; PE, phosphatidylethanolamine; PE(P), phosphatidylethanolamine; PS, phosphatidylserine; other abbreviations as in Figures 1, 2.

We focused on SCAD vs. UA for transition from coronary stability to instability and UA vs. MI for cardiac events. The logistic regression analysis of the metabolic and lipidomic profiles against UA (vs. SCAD) adjusting for traditional risk factors, identified 72 metabolites/lipid species that were significantly (*p* < 0.05) associated with UA (Figure 3A). The regression analysis against MI (vs. UA) conducted by adjusting for traditional risk factors identified 88 metabolites/lipid species that were significantly (*p* < 0.05) associated with MI (Figure 3B). The enrichment pathway analysis of significantly differential metabolites and lipid species for SCAD vs. UA and UA vs. MI are presented in Supplementary Figure S3 and Supplementary Table S10. For SCAD vs. UA, the metabolism pathway significantly changed in glycerophospholipid metabolism (*p* = 7.22E-05, FDR = 6.06E-03) and valine, leucine, and isoleucine biosynthesis (*p* = 1.25E-03, FDR = 5.26E-02). Furthermore, the pathway analysis revealed that cysteine and methionine metabolism (*p* = 4.88E-03, FDR = 0.263) and glycerophospholipid metabolism (*p* = 6.26E-03, FDR = 0.263) were the main perturbed pathways for UA vs. MI. The glycerophospholipid metabolism was the most significantly altered pathway among all paired comparisons.

### Generating Optimal Diagnosis Models for Subgroup Identification and Prediction

**FIGURE 4:**
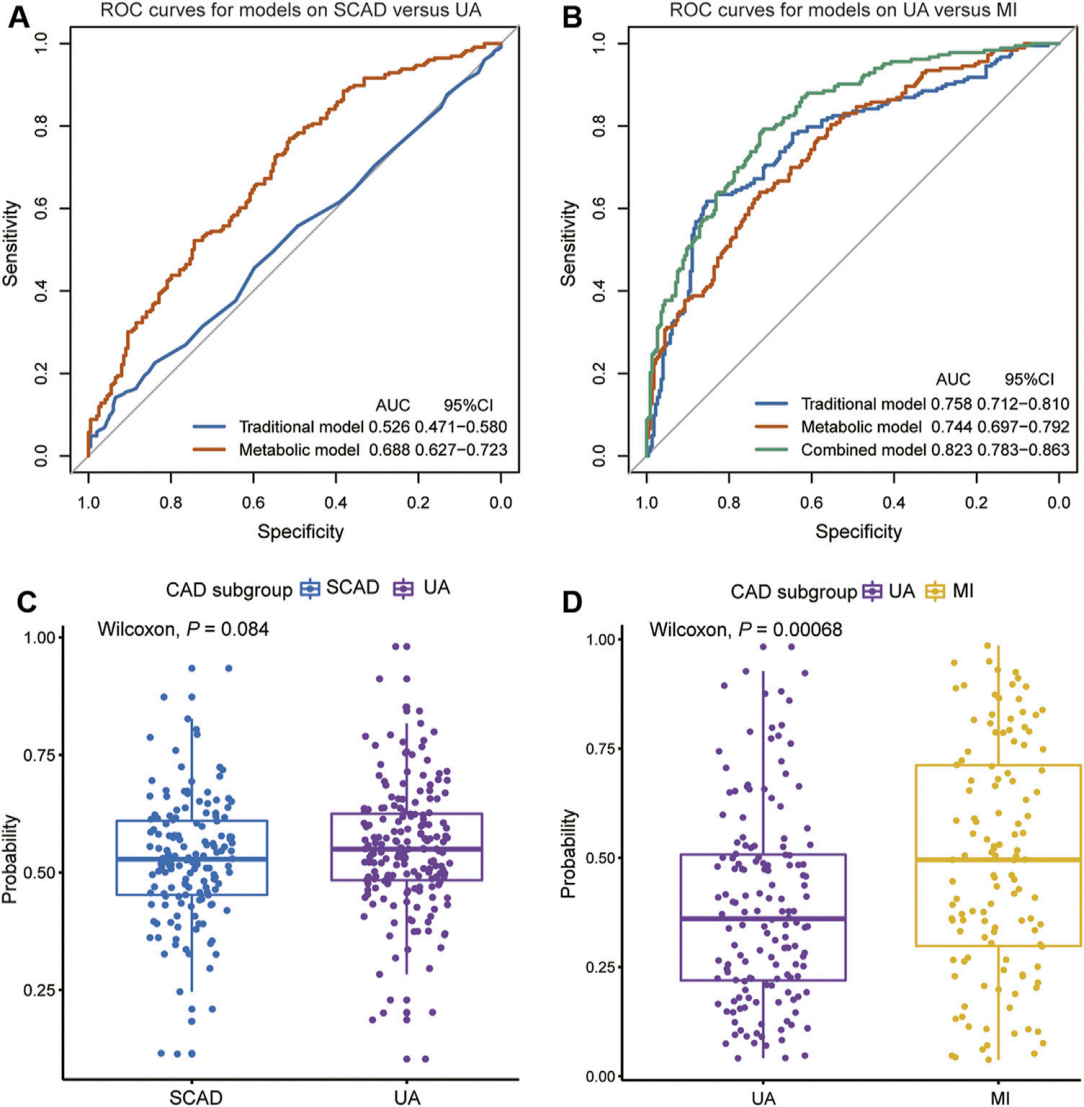
Diagnostic performances in discovery cohort are shown via ROC curves between **(A)** SCAD vs. UA **(B)** UA vs. MI. The combined diagnosis score in validation cohort were compared between **(C)** SCAD and UA patients **(D)** UA and MI patients. AUC, area under curve; CI, confidence interval; ROC, receiver operating characteristic; other abbreviations as in Figure 1.

We focused on UA vs. MI for the prediction of cardiac events. In the first model (the traditional model), 11 conventional CAD risk factors, including age, sex, hypertension, diabetes mellitus, smoking, TG, LDLC, HDLC, APOA, Lp(a), and LVEF, were considered in the stepwise variable selection modeling. Finally five variables, namely, age, hypertension, TG, HDLC, and LVEF were retained in the model with minimal AIC and had an AUC value of 0.758 (Figure 4B; Table 2). In the second model (metabolic model), 88 metabolites/lipid species that were significantly (*p* < 0.05) associated with MI (vs. UA) were considered as input variables. The model was obtained by the LASSO logistic analysis (5-fold cross validation, 200 repeats, Table 3) and consisted of 16 metabolic biomarkers that performed similarly as the traditional model with continuous NRI of −0.0763 (95% CI, −0.271–0.118, *p* = 0.443) and IDI of −0.0178 (95% CI, −0.0669–0.0313, *p* = 0.478; Tables 2, 3). The third model (combined model) incorporated the 11 most predictive metabolic biomarkers to four conventional risk factors from LASSO selection (Table 3). It yielded better discrimination for the prediction of MI than the traditional model with an increased AUC from 0.758 to 0.823 (Figure 4B), a continuous NRI of 0.751 (95% CI, 0.571–0.932, *p* < 0.0001), and an IDI of 0.105 (95% CI, 0.072–0.137, *p* < 0.0001; Table 2).

**TABLE 2:** Model performance measures (95% CIs) for discrimination of CAD subtypes in the discovery cohort.

	Prediction of UA (vs. SCAD)				
Feature	AUC	IDI	*p* Value	Continuous NRI	*p* Value
Traditional model^a^	0.526 (0.471–0.580)				
Metabolic model^b^	0.688 (0.627–0.723)	0.105 (0.0749–0.135)	<0.0001	0.474 (0.289–0.659)	<0.0001
	**Prediction of MI (vs. UA)**				
**Feature**	**AUC**	**IDI**	***p* Value**	**Continuous NRI**	***p* Value**
Traditional model^c^	0.758 (0.712–0.810)				
Metabolic model^d^	0.744 (0.697–0.792)	−0.0178 (−0.0669–0.0313)	0.443	−0.0763 (−0.271–0.118)	0.478
Combined model^e^	0.823 (0.783–0.863)	0.105 (0.072–0.137)	<0.0001	0.751 (0.571–0.932)	<0.0001

^a^ Traditional model for UA (vs. SCAD) based on LVEF.

^b^ Metabolic model for UA vs. SCAD based on: D-Norvaline, LPC(20:5/0:0), HexCer(d18:1/18:1), Cer(m18:1/22:1), 3,3′,5-Triiodo-L-thyronine, LPC(20:0/0:0), D-Sucrose, TG (18:2/18:3/20:2), 3-Hydroxy-3-methyl butyric acid, L-Isoleucine, Deoxycholic acid, 1-Methylxanthine, LPC (16:0/0:0), PC(18:3/20:4), PE (40:3), PC(O-42:5), and TG (14:0/20:3/20:3).

^c^ Traditional model for MI (vs. UA) based on: age, hypertension, TG, HDLC and LVEF.

^d^ Metabolic model for MI (vs. UA) based on: TG (14:0/20:3/22:2), 3-Hydroxy-3-methyl butyric acid, HexCer(d18:1/22:0), HexCer(d18:1/22:1), PA (36:1), PC(O-38:2), D-Methionine, Deoxycholic acid, PC(18:2/18:2), HexCer(d18:1/26:1), PC(O-34:2), PC(18:2/20:2), L-Cystine, TG (14:1/16:1/22:3), 3-Methylcrotonyl glycine, and MG (18:1).

^e^ Combined model for MI (vs. UA) based on: age, LVEF, HexCer(d18:1/22:1), 3-Hydroxy-3-methyl butyric acid, CerP (d18:1/20:3), Cer(d18:1/22:1), PC(18:2/18:2), PC(16:0/20:3), HDLC, Hypertension, PC(18:2/20:4), PC(O-38:2), Deoxycholic acid, L-Cystine, and D-Methionine.

AUC, area under the curve; CAD, coronary artery disease; CI, confidence interval; IDI, integrated discrimination improvement; NRI, net reclassification improvement; other abbreviations as in Tables 1, 4.

**TABLE 3:** Feature inclusion frequency using LASSO based feature selection for MI (vs. UA).

	Metabolites-only model			Metabolites and risk factor model		
	Variable	Frequency	Coefficient	Variable	Frequency	Coefficient
1	TG (14:0/20:3/22:2)	200	0.16	Age	200	−0.02
2	3-Hydroxy-3-methyl butyric acid	200	0.13	LVEF	200	−0.05
3	HexCer(d18:1/22:0)	200	0.08	HexCer(d18:1/22:1)	200	−0.13
4	HexCer(d18:1/22:1)	200	−0.16	3-Hydroxy-3-methyl butyric acid	200	0.19
5	PA (36:1)	200	−0.17	CerP (d18:1/20:3)	199	−0.08
6	PC(O-38:2)	200	−0.22	Cer(d18:1/22:1)	185	−0.05
7	D-Methionine	199	0.12	PC (18:2/18:2)	174	−0.02
8	Deoxycholic acid	199	−0.06	PC (16:0/20:3)	166	0.15
9	PC (18:2/18:2)	199	−0.12	HDLC	149	−0.04
10	HexCer (d18:1/26:1)	182	0.06	Hypertension	126	−0.04
11	PC (O-34:2)	182	−0.03	PC (18:2/20:4)	126	−0.04
12	PC (18:2/20:2)	182	−0.07	PC(O-38:2)	126	−0.02
13	L-Cystine	137	0.02	Deoxycholic acid	126	−0.01
14	TG (14:1/16:1/22:3)	137	−0.02	L-Cystine	101	0.01
15	3-Methylcrotonyl glycine	104	0.02	D-Methionine	101	0.02
16	MG (18:1)	104	−0.05			

LASSO based feature selection was performed within a 5-fold cross-validation framework (200 iterations). Variables selected with frequency >100 times and their average coefficient (Coefficient) were indicated. MI, myocardial infarction; PA, phosphatidic acid; MG, monoglyceride, other abbreviations as in Table 4.

However, the discriminating performance between SCAD and UA was not as satisfactory. The characteristics at baseline of the discovery cohort did not show many differences, and the traditional model based on AIC selection only included LVEF as the predictor with a poor AUC of 0.526. Nevertheless, the utilization of metabolic and lipidomic biomarkers provided another approach for discrimination. On the basis of the 72 variables (*p* < 0.05) selected from adjusted logistic regression, LASSO logistic analyses were further applied to identify the most predictive biomarkers. The optimized model consisting of 17 features exhibited a considerable performance with an AUC of 0.688 (Tables 2, 4). The ROC curve of SCAD vs. UA is plotted in Figure 4A. The diagnostic efficiency of the metabolic model showed a small improvement compared with that of the traditional model with an AUC from 0.562 to 0.688, a continuous NRI of 0.474 (95% CI, 0.289–0.659, *p* < 0.0001), and a IDI of 0.105 (95% CI, 0.0749–0.135, *p* < 0.0001; Table 2).

**TABLE 4:** Feature inclusion frequency using LASSO based feature selection for UA (vs. SCAD).

	Metabolites-only model			Metabolites and risk factor model		
	Variable	Frequency	Coefficient	Variable	Frequency	Coefficient
1	D-Norvaline	195	−0.26	PE (40:3)	70	−0.03
2	LPC (20:5/0:0)	194	−0.08	Cer(m18:1/22:1)	70	0.06
3	HexCer(d18:1/18:1)	194	0.09	Cer(t18:0/24:1)	70	0.01
4	Cer(m18:1/22:1)	191	0.26	HexCer(d18:1/18:1)	70	0.02
5	3,3′,5-Triiodo-L-thyronine	191	−0.13	D-Norvaline	70	−0.05
6	LPC (20:0/0:0)	184	−0.08	LPC (20:5/0:0)	68	−0.01
7	D-Sucrose	184	0.05	CerP (d18:1/20:3)	58	0.01
8	TG (18:2/18:3/20:2)	177	−0.04	PC (16:0/16:1)	47	0.01
9	3-Hydroxy-3-methyl butyric acid	177	0.08	PC (18:3/20:4)	47	0.00
10	L-isoleucine	177	−0.07	LPA (18:1/0:0)	37	0.00
11	Deoxycholic acid	167	0.02	Deoxycholic acid	37	0.00
12	1-Methylxanthine	167	−0.03	1-Methylxanthine	37	0.00
13	LPC (16:0/0:0)	167	−0.05	LPC (16:0/0:0)	37	−0.01
14	PC (18:3/20:4)	153	0.00	D-Sucrose	24	0.00
15	PE (40:3)	153	−0.01	LVEF	8	0.00
16	PC(O-42:5)	153	0.05	L-isoleucine	3	0.00
17	TG (14:0/20:3/20:3)	153	−0.05	HDLC	2	0.00
18				LDLC	2	0.00
19				PE (38:7)	2	0.00
20				PC(O-42:5)	2	0.00

LASSO based feature selection was performed within a 5-fold cross-validation framework (200 iterations). Variables selected with frequency >100 times and their average coefficient (Coefficient) were indicated. Cer, ceramide; HDLC, high-density lipoprotein cholesterol; HexCer, hexosylceramide; LASSO, least absolute shrinkage and selection operator; LDLC, low-density lipoprotein cholesterol; LPA, lysophosphatidic acid; LPC, lysophosphatidylcholine; LVEF, left ventricular ejection fraction; PC, phosphatidylcholine; PC(O), alkylphosphatidylcholine; PE, phosphatidylethanolamine; SCAD, stable coronary artery disease; TG, triacylglycerol; UA, unstable angina.

We subsequently assessed the optimal model for ability to differentiate among CAD subgroups in the validation cohort (Supplementary Table S11). The validation cohort was also divided into the following groups: SCAD (*N* = 152); UA (*N* = 184); and MI (*N* = 157). We used the established optimal LASSO models to further demonstrate the potential ability of subgroup discrimination. Consistently, the combined diagnostic score could help differentiate UA vs. MI patients (*p* < 0.001, Figure 4D). Similarly, the performance on SCAD and UA patients was not satisfactory (*p* = 0.084, Figure 4C).

## Discussion

In this study, we demonstrated that the plasma metabolomic and lipidomic signatures changed dynamically with CAD progression, implying that CAD may involve a universal metabolomic and lipidomic disturbance. A total of 72 and 88 metabolites/lipid species have been identified to be significantly associated with UA (vs. SCAD) and MI (vs. UA), respectively. Moreover, the pathway analysis of these potential biomarkers indicated that glycerophospholipid metabolism exhibited the most significantly altered metabolic pathway in all paired comparisons. Lastly, the newly developed combined diagnostic models improved stratification performance of CAD subtypes compared with the traditional risk model, offering further evidence of dysbiotic metabolome and lipidome and highlighting its potential to distinguish various stages of CAD.

Specifically, the co-clustering modules within lipid classes including phosphatidylcholine (PC), lysophosphatidylcholine (LPC), lysophosphatidylethanolamine (LPE), phosphatidylethanolamine (PE(P)), and alkylphosphatidylcholine (PC(O)) tended to decrease with plaque instability and were inversely correlated with CAD severity and myocardial markers. Moreover, modules containing five PCs were positively correlated with HDLC (as seen in darkolivegreen module, Rho = 0.314, FDR = 5.77E-21) and primarily decreased in the MI group. Different PC species showed diverse effects on CAD progression. We observed that PCs with longer and more unsaturated acyl chain had an inverse association with UA (vs. SCAD). PCs are the most abundant membrane lipids in mammals (van Meer et al., 2008) and are the key structural molecules in the surface monolayer of HDL particles (Kontush et al., 2013). Shorter and highly saturated acyl chains of PC molecules confer less fluidity of the lipid monolayer, thereby directly affecting HDL’s ability to accept cholesterol from peripheral tissues and phospholipid hydroperoxides from low-density lipoproteins (Kontush et al., 2013; Toledo et al., 2017).

PC in lipoproteins or from cell membrane can be further hydrolyzed on the sn-2 position fatty acid to generate LPC and free fatty acid by the phospholipase A2 enzyme (Norris et al., 2014). Although the catalysis of phospholipase A2 was expected to generate LPC to promote inflammation and atherosclerosis development (Huang et al., 2020; Schmitz and Ruebsaamen, 2010), most LPC species exhibited a negative association with UA (vs. SCAD) and MI (vs. UA). As shown in Figure 3, LPC(16:0/0:0), LPC(20:0/0:0), and LPC(20:5/0:0) were decreased in UA patients compared with SCAD patients. LPC(22:0/0:0) and LPC(18:2/0:0) were decreased in MI patients (vs. UA), which is consistent with previously reported results (Fan et al., 2016; Lu et al., 2017). LPC is reportedly an inducer of endothelial dysfunction and a regulator of vascular tone (Zhang et al., 2009; Paapstel et al., 2018). Lower levels of LPC in the circulation may result from the increase in the catabolism of these species or to their more efficient removal from blood circulation into the tissues, either in the form of modified lipoprotein or directly from albumin (Meikle et al., 2011).

One of the prominent features observed was that a number of PE(P) species with polyunsaturated fatty acids displayed a significant inverse association with MI compared with UA patients (Figure 3B). Alkylphospholipids [alkylphosphatidylcholine, PC(O) and alkylphosphatidylethanolamine, PE(O)] and alkenylphospholipids [primarily presented as phosphatidylcholine, PC(P), and phosphatidylethanolamine, PE(P) species, equivalently known as plasmalogens] have been proposed to protect against atherosclerosis due to their antioxidant characteristics and a high proportion of polyunsaturated fatty acids and alkyl/alkenyl linked fatty acids. They are more susceptible to oxidation under heightened oxidative stress (Lessig and Fuchs, 2009; Ford, 2010). In addition, plasmalogens are essential for intracellular cholesterol transport (Munn et al., 2003) and HDLC-mediated cholesterol efflux (Maeba et al., 2018). Recently, the inclusion of plasmalogens into reconstituted HDL improved the lipoprotein anti-apoptotic activity on endothelial cells (Sutter et al., 2015). Therefore, low plasmalogens levels in plasma may reflect the high oxidative stress and the action of reactive oxygen species on these lipids.

However, the module containing ceramides was elevated with disease shifting and was positively associated with CAD severity, myocardial markers, and inflammatory state. Notably, hexosylceramide species played an important role in the development of CAD. Specific hexosylceramide species [e.g., HexCer(22:0/0:0)] were related to the enhanced coronary atherosclerosis burden. Both mono- and dihexosylceramide have a direct association with the risk of future cardiovascular events in patients with type 2 diabetes, which is a potential atherogenesis-contributing factor (Alshehry et al., 2016).

Some plasma ceramide levels were observed to be up-regulated with the disease shifting direction as SCAD, UA, and MI and positively correlated with atherosclerosis burden quantified by the SYNTAX score and SYNTAX score Ⅱ and the evidence of subclinical myonecrosis quantified by cTnI. This result corroborates the finding that elevated plasma ceramide levels are independent biomarkers of MACE (Laaksonen et al., 2016). Cer (d18:1/20:1) was significantly elevated in UA (vs. SCAD) and we previously reported that Cer (d18:1/20:1) was negatively related with LVEF and could serve as an independent predictor of MACE and all-cause mortality (Qin et al., 2020). As the metabolites of sphingolipid, ceramides are considered lipotoxic inducers of disturbed glucose homeostasis and insulin resistance and causative agents in the pathophysiology of atherosclerosis (Chaurasia and Summers, 2015; Laaksonen et al., 2016). Studies in rodent models revealed that the inhibition of ceramide synthesis prevents ischemic cardiomyopathy-related heart failure post hypoxia or MI while simultaneously diminishing ventricular remodeling and lowering cell death rates and changing the abundance of proinflammatory detrimental neutrophils (Park and Goldberg, 2012; Hadas et al., 2020). The underlying functions of ceramides involve the promotion of lipoprotein transport into the arterial wall, platelet activation, and endothelial dysfunction via uncoupling of NO signaling pathways (Chaurasia and Summers, 2015; Meikle and Summers, 2017).

In addition to the use of certain lipid species of sphingolipids and glycerophospholipids as predictors of CAD progression, the downregulation of deoxycholic acid in MI, which plays key roles in bile acid and cholesterol metabolism, indicated that the metabolism of cholesterol and phospholipids might be inhibited (Sayin et al., 2013). Moreover, low triiodothyronine was inversely associated with UA occurrence, which indicated a close link between thyroid function and atherosclerosis process. Since triiodothyronine is the most biologically active thyroid hormone, it plays a vital role in regulating heart rate, contractile force, and peripheral arterial resistance (Jabbar et al., 2017). A meta-analysis of 56 studies showed that a reduced serum triiodothyronine level was further associated with the increased risk of all-cause and cardiogenic death, and was an independent predictor of MACE (Wang B. et al., 2017). Lastly, a number of amino acids and their derivatives were altered with CAD shifting. Elevated levels of plasma cystine (the disulphide form of cysteine) were positively associated with MI (vs. UA) and werepositively correlated with SYNTAX score Ⅱ and hs-CRP, which is indicated to link with a higher oxidative stress and endothelial dysfunction (Oliveira and Laurindo, 2018). A high level of methionine served as a strong predictor for MI (vs. UA) selected by LASSO. A previous study has shown that methionine promotes atherosclerotic plaques independent of homocysteine levels in the rodent model (Selhub and Troen, 2016).

Our study had some limitations that needed be considered. First, due to the upgrading of analytical platform and technical issues with the mass spectrometry, the metabolites and lipid species detected were not in accordance, thereby resulting in the lack of four independent predictors for UA (vs. SCAD) model and one for MI (vs. UA) model which affected the model estimation in the verification cohort. Second, site-to-site and observer-to-observer variations in the evaluation of coronary stenosis may exist, leading to diagnostic bias. Third, the improvement in AUC for a model is often very minor, yet the category-free NRI may overstate the incremental value of a biomarker. Last, our study population tended to consist of middle-aged to elderly Chinese patients. Thus, other ethnicities within Asia and other races, such as Caucasians and Africans, should be included in future studies.

## Conclusion

Multiple plasma metabolites and lipid species differed between CAD subgroups, and the alterations were correlated with CAD severity. The metabolites involved in glycerophospholipid metabolism appeared to be a predominant alteration in CAD progression. A small number of these biomarkers significantly improved the diagnostic value for differentiating patients between CAD types. These findings may help to predict disease progression and clinical outcome and indicate the potential for novel intervention strategies to attenuate disease progression.

## Data Availability

The raw data supporting the conclusions of this article will be made available by the authors, without undue reservation.
