# Supplementary material for: Comprehensive Metabolomics Identified the Prominent Role of Glycerophospholipid Metabolism in Coronary Artery Disease Progression
Source: Front Mol Biosci. 2021 Apr 14;8:632950. doi: 10.3389/fmolb.2021.632950 (PMC8080796; doi:10.3389/fmolb.2021.632950)
Supplement: Supplementary file 1 [file datasheet1.docx]

***Supplementary Material***

**Comprehensive metabolomics identified the prominent role of glycerophospholipid metabolism in coronary artery disease progression**

**Hui Chen ^1, 2, 3 #^, Zixian Wang ^1, 2, 3, 4 #^, Min Qin ^1, 2, 3^, Bin Zhang ^3, 5^, Lu Lin ^2^, Qilin Ma ^6^, Chen Liu ^7^, Xiaoping Chen ^8^, Hanping Li ^3^, Weihua Lai ^2^, Shilong Zhong^1, 2, 3, 4*^**

^#^These authors contributed equally to the study.

***Correspondence:**

Shi-Long Zhong: [gdph_zhongsl@gd.gov.cn](mailto:gdph_zhongsl@gd.gov.cn)

**Supplementary Methods**

**Criteria of CAD subgroups**

The diagnosis of SCAD is based on substernal chest discomfort with a characteristic quality and duration that is provoked by exertion or emotional stress and can be relieved by rest or nitroglycerin(1).UA is defined as with a normal cardiac troponin measurement and at least one of the following presentations: (1) rest angina, occurred at rest and prolonged >20min, occurring within 1 week of presentation. (2) new-onset angina, angina of at least Class Ⅲ severity according to the Classification of the Canadian Cardiovascular Society with onset within 2 months of initial presentation. (3) increasing angina, recent destabilization (that is distinctly more frequent, longer in duration, or lower in threshold) of previously diagnosed angina with at least Canadian Cardiovascular Society Class III angina characteristics(2). MI is defined as a rise and/or fall of cardiac biomarker values (preferably cardiac troponin) with at least one value above the 99^th^ percentile upper reference limit and with at least one of the following: (1) ischaemia symptoms; (2) new or presumed new significant ST-segment-T wave changes or new left bundle branch block; (3) development of pathological Q waves in the electrocardiogram; (4) imaging evidence of new viable myocardium loss or new regional wall motion abnormality; (5) identification of an intracoronary thrombus by angiography or autopsy(3).

**SYNTAX score systems**

We used the Synergy between PCI with TAXUS and Cardiac Surgery (SYNTAX) score, an angiographic scoring system to determine the complexity, severity, and atherosclerotic burden of CAD(4). The SYNTAX score was calculated for each patient using a computer program that consisted of sequential and interactive self-guided questions according to the SYNTAX score calculator version 2.11 (http://www.syntaxscore.com/). The SYNTAX score characterizes the anatomy of coronary vasculature with respect to the lesion number, lesion location, the occurrence of total occlusions, bridging collaterals, bi/trifurcations, aorto-ostial, tortuosity, lesion length, calcification, thrombus and diffuse disease/small vessels. However, due to some limitations of this score, including lacking of clinical variables and a purely anatomical focus, the SYNTAX score Ⅱ was developed with a presumed improved prognostic value.

The SYNTAX score Ⅱ were also calculated by using patients’ baseline clinical characteristics as previously described(5). In Brief, SYNTAX score Ⅱ (<http://www.syntaxscore.com/calculator/syntaxscore/framesetss2.htm>) was computed by an online pre-defined algorithm, based on the SYNTAX score and unprotected left main CAD, other clinical variables were added taking into account [age, sex, left ventricular ejection fraction, creatinine clearance (in millimeters per min), chronic obstructive pulmonary disease, and peripheral vascular disease], leading to the SYNTAX score Ⅱ.

**Sample preparation and extraction**

For metabolic compounds profiling, plasma sample was thawed on ice, 150 μL chilled methanol was added to 50 μL of each plasma for protein precipitation. The mixture was vortexed for 3 min and then centrifuged with 12,000 rpm at 4 ℃ for 10 min. The supernatant was transferred into another clean EP tube and centrifuged again for 5 min under the same conditions as the first time, and the supernatant after the second centrifugation was used for the following UPLC-MS/MS analysis.

For lipids profiling, the thawed sample was gently vortexed for 10s, and then centrifuged with 3000 rpm at 4 ℃ for 5 min. 50μL of each sample was homogenized with 1mL mixture (include methanol, MTBE and internal standard mixture), vortexed for 2 min, followed by the addition of 500 μL pure water, vortexed for 1min again, then centrifuged with 12,000 rpm at 4℃ for 10 min. 500 μL of supernatant was removed and dried. The residue was reconstituted in 100 μL of mobile phase B (seen as UPLC Conditions) and subsequently analyzed by UPLC-MS/MS system.

**UPLC Conditions**

Chromatographic analysis was performed using UPLC system (Shim-pack UFLC SHIMADZU CBM30A). For metabolic profiling (hydrophilic compounds), chromatographic separation was performed on the Waters ACQUITY UPLC HSS T3 reversed-phase column(1.8μm, 2.1mm×100mm) with the mobile phases A of 0.04% acetic acid (Fisher Scientific) in water (Millipore) (*v/v*) and B of 0.04% acetic acid in acetonitrile (Fisher Scientific) (*v/v*) at column temperature of 40℃ and gradient flow rate of 0.4 mL/min. Gradient elution was programmed as follows: 0-11.0 min increased from 5% to 95% B and maintained at 95% B for 1min,12-12.1 min decreased from 95% to 5% B and maintained to 14.0 min. The injection volume was 5 μL.

While under lipidic profiling (hydrophobic compounds), chromatographic separation was performed on the Thermo C30 column (2.6μm, 2.1mm×100mm) with the mobile phases A of 0.04% acetic acid and 5 mM ammonium formate in acetonitrile/water (60:40, *v*:*v*) and mobile phase B of 0.04% acetic acid and 5mM ammonium formate in acetonitrile/isopropanol (10:90, *v:v*) at 45 ℃ and a flow rate of 0.35 mL/min. Gradient program was as follows: 0-3min increased from 20% to 50% B, 3-5min increased from 50% to 65% B, 5-9 min increased from 65% to 75% B, 9-15.5 min increased from 75% to 90% B. The injection volume was 2 μL.

**ESI-QTRAP-MS/MS Conditions**

After separation by UPLC, mass spectrometry was performed using a triple quadrupole-linear ion trap (QTRAP), equipped with an electrospray ionization ESI Turbo Ion-Spray interface (AB Sciex). LIT and triple quadrupole (QQQ) scans were acquired on a triple quadrupole-linear ion trap mass spectrometer (QTRAP). Except Applied Biosystems 4500 QTRAP was utilized for hydrophilic compounds profiling in discovery set, all the measurements were conducted by QTRAP® 6500+ LC-MS/MS System, equipped with an ESI Turbo Ion-Spray interface, operating in positive and negative ion mode and controlled by Analyst 1.6.3 software (AB Sciex).

The ESI source operation parameters were as follows: ion source, turbo spray; source temperature 550 °C; ion spray voltage (IS) 5.5kV under positive ion mode(or -4.5kV in negative ion mode); ion source gas I (GSI), gas II (GSII), curtain gas (CUR) were respectively set at 55, 60, and 25 psi; the collision gas (CAD) was medium. Instrument tuning and mass calibration were performed with 10 and 100 μmol/L polypropylene glycol solutions in QQQ and LIT modes, respectively. QQQ scans were acquired as MRM experiments with collision gas (nitrogen) set to 5 psi. Declustering potential (DP) and collision energy (CE) for individual MRM transitions was done with further DP and CE optimization. A specific set of MRM transitions were monitored for each period according to the compounds eluted within this period.

**Plasma Metabolites and lipids qualitative and quantitative analysis**

Qualitative analysis of the precursor ion and fragments spectra detected was carried out on the basis of [self-built](javascript:;) MWDB (metware database) with retention time and ion pairs, as well as the public database of metabolites information. We used MS/MS spectra to search against public databases to improve confidence in metabolite identification. Some of these substances are qualitatively analyzed with removing isotopic signals, repetitive signals containing K^+^ ions, Na^+^ ions, and NH4^+^ ions, as well as repeated signals of fragmented ions that themselves are of larger molecular weight. Metabolite and lipid structure resolution is referenced in existing mass spectrometry public databases such as MassBank (http://www.massbank.jp/)(6), HMDB (http://www.hmdb.ca/)(7), METLIN (http://metlin.scripps.edu/index.php)(8) and the Lipid maps Structure Database (LMSD, https://www.lipidmaps.org/data/structure/)(9). The metabolite identification was conducted by alignment to the reference standards in our [self-built](javascript:;) database and public databases, and more information is listed in Table S1-S2. MetaboAnalyst (https://www.metaboanalyst.ca) (version 4.0)(10) and Kyoto Encyclopedia of Genes and Genomes (KEGG) database (http://www.genome.jp/kegg/) (11)was used to analyze the pathway enrichment for the identification of highly enriched metabolic pathways in differential metabolites or lipids. The *P*-value <0.05 was considered as the significantly changing pathways.

Quantitation of metabolites was accomplished using multiple reaction monitoring (MRM) of triple quadrupole mass spectrometry. Under the MRM mode, the quadrupole rod first screened precursor ions (parent ions) of the target substance to exclude ions corresponding to other molecular weight substances to preliminarily eliminate the interference. The precursor ions were induced to ionize in the collision cell to form many fragment ions, fragment ions. The fragment ions are then filtered through the triple four-stage bar to select a characteristic fragment ion needed, which eliminates the interference of non-target ions, making the quantification of better accuracy and repeatability. The mass spectrum data were processed by Analyst 1.6.3 software (AB Sciex). After obtaining the metabolite spectrum analysis data of different samples, the peak area integral was performed for the mass spectrum peaks, and the integral correction was performed for the mass spectrum peaks of the same metabolite in different samples.

**Quality control**

Quality control (QC) samples were prepared with pooling aliquots of each of the individual plasma samples, which were extracted as described above. During instrumental running, one QC sample was inserted into every 10 samples to monitor the analysis reproducibility. Extracts of this pooled plasma sample were injected to access process variability. As an additional QC, water aliquots were extracted as part of the sample set to serve as process blanks for artifact identification. By overlapping display analysis of the total ion chromatogram (TIC) of different QC samples for mass spectrometric detection and analysis, the repeatability of the extraction and detection of metabolites can be judged, that is, technical repeatability. The high stability of the instrument provides an important guarantee for the repeatability and reliability of the data (Figure S1).

**References**

1. Fihn SD, Gardin JM, Abrams J, Berra K, Blankenship JC, Dallas AP, et al. 2012 ACCF/AHA/ACP/AATS/PCNA/SCAI/STS Guideline for the diagnosis and management of patients with stable ischemic heart disease: a report of the American College of Cardiology Foundation/American Heart Association Task Force on Practice Guidelines, and the American College of Physicians, American Association for Thoracic Surgery, Preventive Cardiovascular Nurses Association, Society for Cardiovascular Angiography and Interventions, and Society of Thoracic Surgeons. *J Am Coll Cardiol* (2012) 60(24):e44-e164. Epub 2012/11/28. doi: 10.1016/j.jacc.2012.07.013. PubMed PMID: 23182125.

2. Braunwald E. Unstable angina. A classification. *Circulation* (1989) 80(2):410-4. Epub 1989/08/01. doi: 10.1161/01.cir.80.2.410. PubMed PMID: 2752565.

3. Thygesen K, Alpert JS, Jaffe AS, Simoons ML, Chaitman BR, White HD, et al. Third universal definition of myocardial infarction. *Eur Heart J* (2012) 33(20):2551-67. Epub 2012/08/28. doi: 10.1093/eurheartj/ehs184. PubMed PMID: 22922414.

4. Sianos G, Morel MA, Kappetein AP, Morice MC, Colombo A, Dawkins K, et al. The SYNTAX Score: an angiographic tool grading the complexity of coronary artery disease. *EuroIntervention : journal of EuroPCR in collaboration with the Working Group on Interventional Cardiology of the European Society of Cardiology* (2005) 1(2):219-27. Epub 2005/08/01. PubMed PMID: 19758907.

5. Farooq V, van Klaveren D, Steyerberg EW, Meliga E, Vergouwe Y, Chieffo A, et al. Anatomical and clinical characteristics to guide decision making between coronary artery bypass surgery and percutaneous coronary intervention for individual patients: development and validation of SYNTAX score II. *The Lancet* (2013) 381(9867):639-50. doi: 10.1016/s0140-6736(13)60108-7.

6. Horai H, Arita M, Kanaya S, Nihei Y, Ikeda T, Suwa K, et al. MassBank: a public repository for sharing mass spectral data for life sciences. *J Mass Spectrom* (2010) 45(7):703-14. Epub 2010/07/14. doi: 10.1002/jms.1777. PubMed PMID: 20623627.

7. Wishart DS, Feunang YD, Marcu A, Guo AC, Liang K, Vazquez-Fresno R, et al. HMDB 4.0: the human metabolome database for 2018. *Nucleic Acids Res* (2018) 46(D1):D608-D17. Epub 2017/11/16. doi: 10.1093/nar/gkx1089. PubMed PMID: 29140435; PubMed Central PMCID: PMCPMC5753273.

8. Smith CA, O'Maille G, Want EJ, Qin C, Trauger SA, Brandon TR, et al. METLIN: a metabolite mass spectral database. *Ther Drug Monit* (2005) 27(6):747-51. Epub 2006/01/13. doi: 10.1097/01.ftd.0000179845.53213.39. PubMed PMID: 16404815.

9. Sud M, Fahy E, Cotter D, Brown A, Dennis EA, Glass CK, et al. LMSD: LIPID MAPS structure database. *Nucleic Acids Res* (2007) 35(Database issue):D527-32. Epub 2006/11/14. doi: 10.1093/nar/gkl838. PubMed PMID: 17098933; PubMed Central PMCID: PMCPMC1669719.

10. Chong J, Soufan O, Li C, Caraus I, Li S, Bourque G, et al. MetaboAnalyst 4.0: towards more transparent and integrative metabolomics analysis. *Nucleic Acids Research* (2018) 46(W1):W486-W94. doi: 10.1093/nar/gky310.

11. Kanehisa M, Goto S. KEGG: kyoto encyclopedia of genes and genomes. *Nucleic Acids Res* (2000) 28(1):27-30. Epub 1999/12/11. doi: 10.1093/nar/28.1.27. PubMed PMID: 10592173; PubMed Central PMCID: PMCPMC102409.


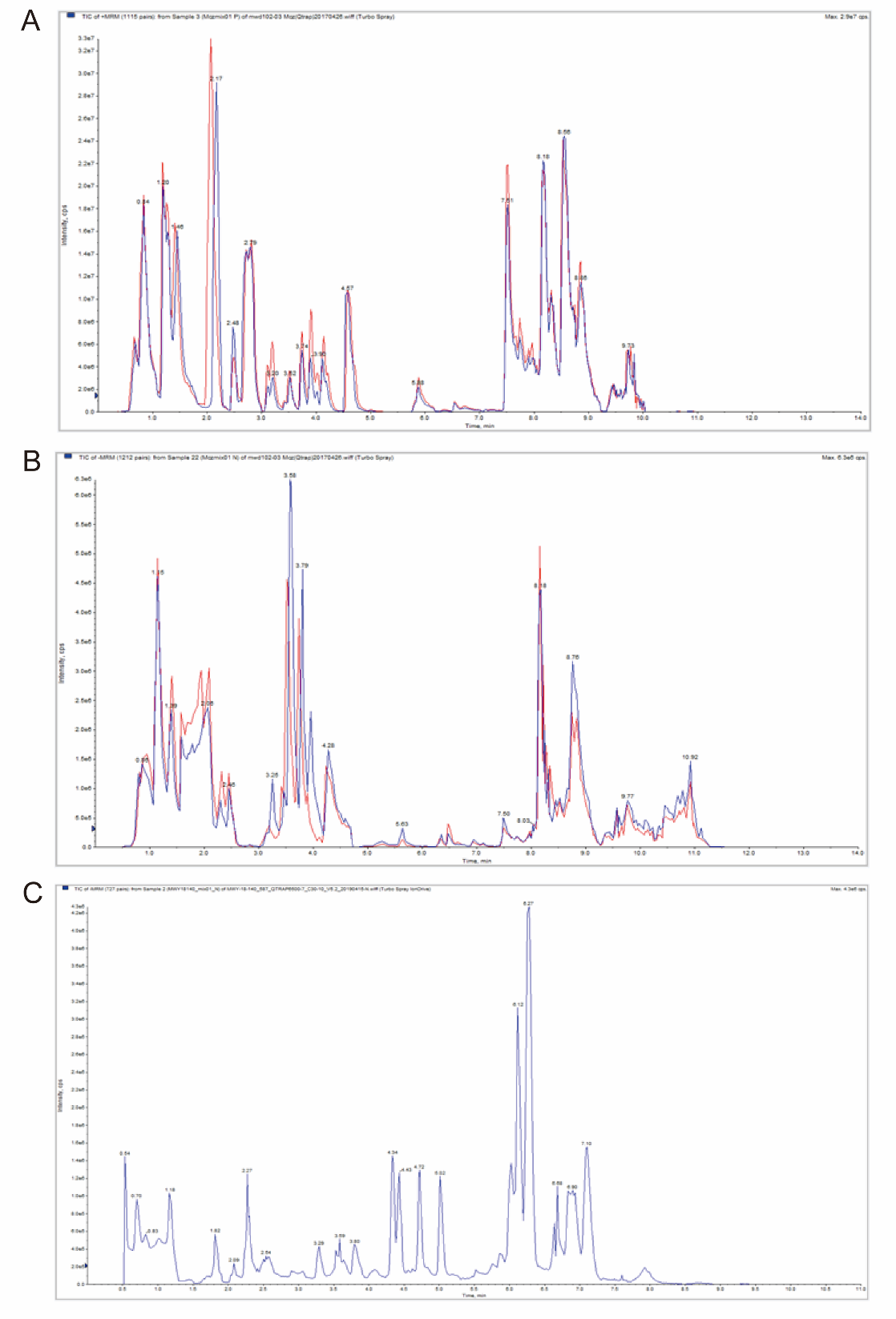


**Figure S1.** Representative total ion flow diagrams between different QC samples. **A** and **B** showed metabolic profiling under positive ion mode and negative ion mode, respectively; **C** presented lipid profiling under positive mode.


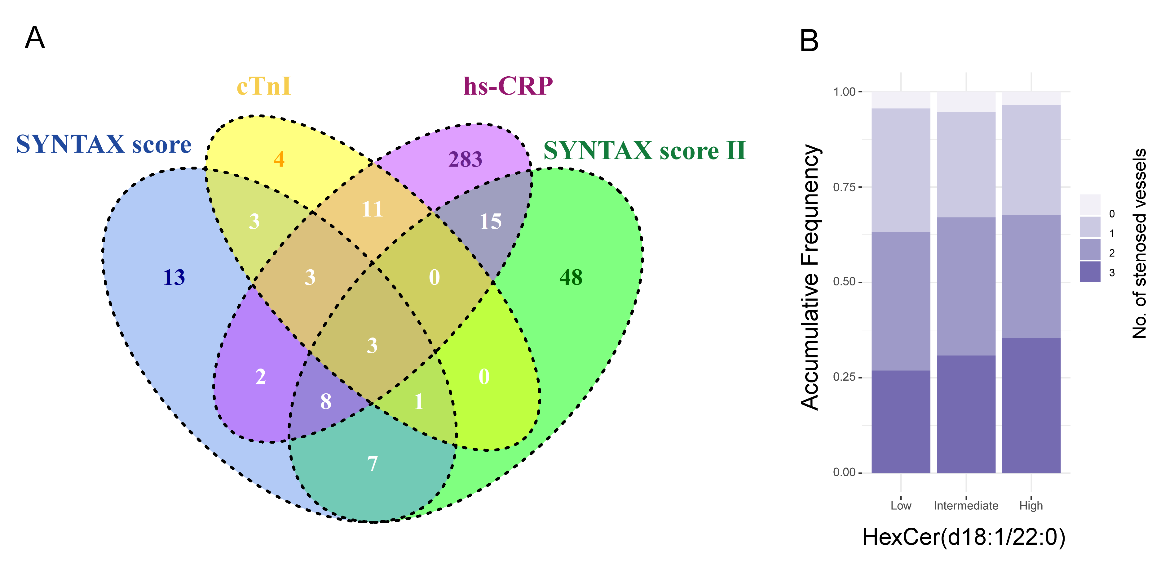


**Figure S2.** Differential metabolites with severity indicators. **(A)** [Venn](javascript:;) [diagram](javascript:;) represented intersection of metabolites associated with different CAD severity indicators. **(B)** Bar plot of HexCer (d18:1/22:0) associated with number of stenosed vessels. Each bar shows the proportion of harboring different number of stenosed vessels in each genotype (the darker the color of the block, the more stenosed vessels it has). Plot indicates that individuals carrying higher tertile of HexCer (d18:1/22:0) are likely to be harboring more stenosed vessels.


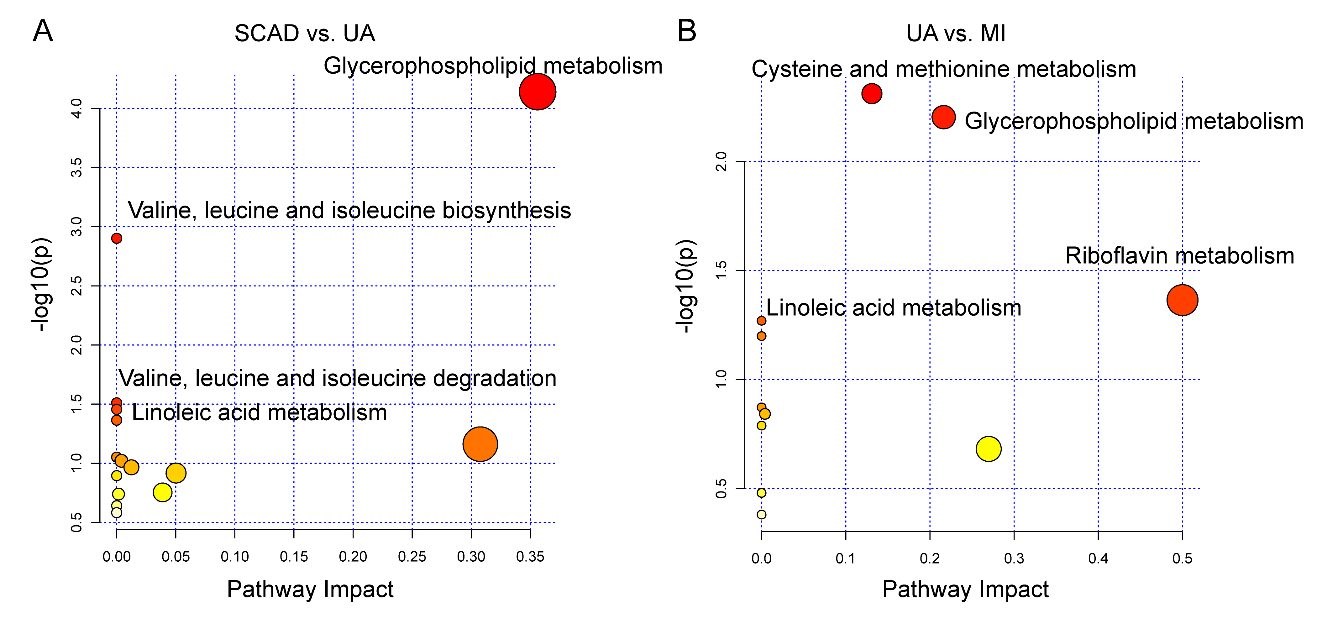


**Figure S3.** The disturbed pathways showed various metabolism changed. (A) SCAD vs. UA, (B) UA vs. MI.
